# An integrated network toxicology and multi-omics study identifies ENO1 as a candidate mediator in benzo[a]pyrene-related gastric cancer progression

**DOI:** 10.3389/fphar.2026.1857843

**Published:** 2026-06-29

**Authors:** Haixia Liu, Binru Di, Xiaoding Men, Jingzhou Zhu, Yaowen Liu, Jing Luo, Jianmei Wang, Li Xiang, Yuhao Luo

**Affiliations:** 1 Department of Oncology, The Affiliated Hospital of Southwest Medical University, Luzhou, China; 2 Department of Cardiology, The Affiliated Hospital of Southwest Medical University, Luzhou, China; 3 Department of Pathology, The Affiliated Hospital of Southwest Medical University, Luzhou, China; 4 Luzhou Key Laboratory of Molecular Cancer, Luzhou, Sichuan, China

**Keywords:** alpha-enolase (ENO1), benzo[a]pyrene, gastric cancer, machine learning, network toxicology, tumor microenvironment

## Abstract

This study employed an integrative toxicogenomic and multi-omics prioritization approach, combined with preliminary *in vitro* validation, to identify candidate mediators of benzo [a]pyrene (BaP)-associated gastric cancer progression. A BaP-target-gastric cancer interaction network was constructed by integrating toxicogenomic databases with public transcriptomic datasets. Candidate genes were prioritized using differential expression analysis, network-based screening, LASSO regression, and exploratory artificial neural network modeling. Molecular docking was used to generate an *in silico* structural hypothesis for possible BaP-ENO1 protein-level interaction, while GO/KEGG enrichment and single-cell RNA sequencing characterized associated biological processes and cellular expression patterns. Integrated analyses prioritized ENO1 as the top BaP-associated candidate in gastric cancer. Single-cell analysis revealed elevated ENO1 expression in gastric cancer epithelial cells and tumor-associated macrophages. *In vitro* experiments demonstrated that BaP enhanced gastric cancer cell proliferation and migration, effects that were partially attenuated by ENO1 knockdown. Collectively, these findings position ENO1 as a candidate mediator of BaP-associated gastric cancer progression and provide preliminary support for further investigation of the BaP-ENO1 relationship. The study also offers a cross-platform framework for prioritizing candidate targets in environmentally linked cancers.

## Graphical Abstract

**Figure:**
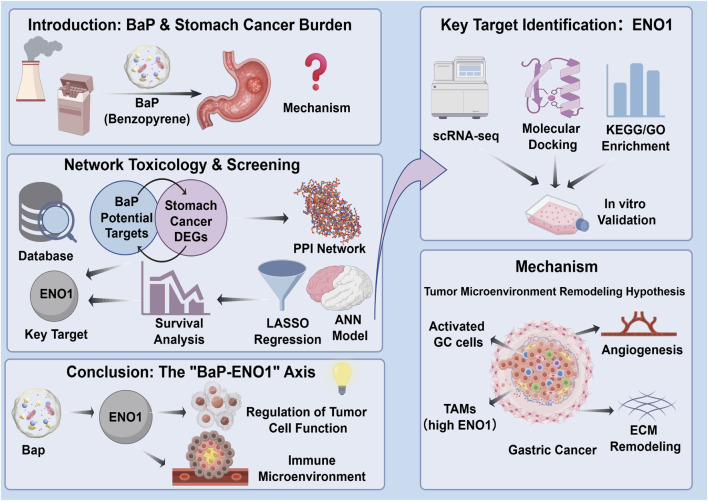

## 1 Introduction

Gastric cancer remains a leading cause of cancer-related morbidity and mortality worldwide (Morgan et al., 2022), characterized by multifactorial and multistage carcinogenesis with high heterogeneity (Mamun et al., 2024). Despite advances in diagnosis and treatment, the 5-year survival rate for advanced-stage patients remains unsatisfactory. In addition to established infectious agents such as *Helicobacter pylori*, widespread environmental and lifestyle exposures-including dietary habits, smoking, and specific pollutants-are recognized as significant risk factors influencing gastric cancer initiation, progression, and therapeutic response (Pan et al., 2024; Karimi et al., 2014). Among these, polycyclic aromatic hydrocarbons (PAHs) have been implicated in gastric carcinogenesis; however, the detailed molecular network through which they drive tumor progression remains largely unelucidated (Blechter et al., 2025). Deciphering the complexity of such gene-environment interactions represent a major challenge in current research. A systematic investigation into the mechanisms of specific environmental carcinogens is therefore imperative to advance our understanding of gastric cancer etiology, improve risk stratification, and inform the development of novel prevention and treatment strategies.

Benzo [a]pyrene (BaP), a representative member of the polycyclic aromatic hydrocarbon (PAH) family, is a ubiquitous environmental pollutant generated from the incomplete combustion of organic materials, including tobacco smoke, industrial emissions, and residential heating (Boström et al., 2002). It is also recognized that certain cooking processes can lead to contamination, including charcoal barbecuing, cooking oil fumes, and the consumption of fatty foods and red meat. Notably high BaP levels have been detected in smoked dried beef (5.47 mg/kg), fried chicken (5.25–5.55 mg/kg), and potato chips (4.06 mg/kg) (Zheng et al., 2022). BaP, a Group 1 human carcinogen as classified by the International Agency for Research on Cancer (IARC) and a priority carcinogen listed by the U.S. Environmental Protection Agency (EPA) (Barbosa et al., 2023), has been epidemiologically linked to an increased risk of gastric cancer in recent population studies (Kim et al., 2013). Experimental evidence further confirms its ability to induce gastric precancerous lesions and tumors *in vivo* (Chemical agents and related occupations, 2012). Although studies have implicated specific pathways, such as the aryl hydrocarbon receptor (AhR) axis, in BaP-driven proliferation and metastasis of gastric cancer cells (Wei et al., 2016), the comprehensive molecular network through which BaP promotes gastric carcinogenesis remains incompletely mapped. In particular, a systematic atlas of its potential molecular targets and their crosstalk with the tumor microenvironment (TME) is poorly understood.

Network toxicology, integrating bioinformatics and systems biology, offers a robust framework for systematically deciphering the complex toxicological mechanisms of environmental chemicals such as BaP at a systems level (Huang, 2024). This strategy enables the systematic prediction and identification of key molecular nodes by constructing comprehensive chemical-target-disease interaction networks. As an integral component of this framework, molecular docking simulations model the binding interactions between a chemical and potential target proteins at an atomic level, providing a structural rationale for critical interactions (Morris and Lim-Wilby, 2008). Concurrently, the integration of genomics with machine learning approaches, including artificial neural networks, facilitates the effective mining of key molecular features associated with patient prognosis, thereby enabling the construction of clinically predictive models (Ching et al., 2018). Together, these methodologies constitute a cohesive, translational research strategy that bridges *in silico* prediction with clinical relevance.

Therefore, this study aimed to apply an integrative strategy combining network toxicology, machine learning, molecular docking, single-cell transcriptomics, and initial *in vitro* assays to prioritize candidate BaP-associated targets in gastric cancer and to provide preliminary biological support for the involvement of ENO1 in BaP-related malignant phenotypes.

## 2 Materials and methods

### 2.1 Integrated multi-omics target identification and network construction

#### 2.1.1 Identification of gastric cancer differentially expressed genes (DEGs)

The gastric cancer transcriptomic dataset GSE118916 was downloaded from the GEO database. Data normalization and differential expression analysis were performed using the limma package in R 4.3.0. Genes with an absolute log2-fold change (|log_2_FC|) > 1 and a Benjamini-Hochberg adjusted p-value <0.05 were defined as DEGs. The results were visualized using a heatmap (pheatmap package) and a volcano plot (ggplot2 and ggrepel packages).

#### 2.1.2 Prediction of potential BaP targets

The SMILES identifier for Benzo [a]pyrene (BaP) was obtained from PubChem and submitted to the Comparative Toxicogenomics Database (CTD) (https://ctdbase.org/). Target genes with an Interaction Count ≥5 were retrieved as potential BaP-binding targets.

#### 2.1.3 Intersection target identification and protein-protein interaction (PPI) network construction

Gastric cancer DEGs, CTD-predicted BaP targets, and gastric cancer-related targets from GeneCards (filtered by a Relevance Score ≥10) were integrated. The intersection of these three sets was defined as the candidate target gene set for downstream prioritization. We note that this intersection-based strategy was used as a heuristic approach for candidate selection and may preferentially retain genes with higher annotation density across curated resources. This candidate set was imported into the STRING database (https://string-db.org/) to construct a PPI network, which was visualized and analyzed using Cytoscape 3.9.1. Core hub nodes within the network were identified using the CytoHubba plugin.

### 2.2 KEGG pathway enrichment analysis

KEGG (Kyoto Encyclopedia of Genes and Genomes) pathway enrichment analysis for the intersecting gene set was performed using R packages including “clusterProfiler,” “org.Hs.eg.db,” “enrichplot,” and “ggplot2.” Specifically, gene symbols were first converted to Entrez IDs. The enrichKEGG function was then applied to perform the enrichment analysis. The resulting enrichments were subsequently mapped back to gene symbols using the setReadable function. P values were adjusted using the Benjamini-Hochberg method, and pathways with an adjusted P value <0.05 were considered significantly enriched.

### 2.3 GO functional enrichment analysis

Gene Ontology (GO) functional enrichment analysis of the intersecting gene set was performed using R packages including “clusterProfiler,” “org.Hs.eg.db,” “ComplexHeatmap,” “RColorBrewer,” and “dplyr.” The analysis encompassed all three main GO categories: Biological Process (BP), Cellular Component (CC), and Molecular Function (MF). Gene symbols were converted to Entrez IDs, enrichment analysis was performed using the enrichGO function, and P values were adjusted using the Benjamini-Hochberg method. GO terms with an adjusted P value <0.05 were considered significantly enriched.

### 2.4 Identification and validation of core genes

#### 2.4.1 Screening of key genes via LASSO regression

Key genes were screened from the candidate gene expression matrix using least absolute shrinkage and selection operator (LASSO) regression implemented with the R package “glmnet”. Data preprocessing included reading the gene expression matrix and transposing it to a format where rows represented samples and columns represented genes. Sample group labels were extracted as a binary response variable, with the gene expression values serving as the input feature matrix. A binary classification LASSO regression model was constructed using the glmnet function. The optimal penalty parameter (lambda) was determined via 10-fold cross-validation (lambda.min). Genes with non-zero coefficients under this optimal lambda were identified as key genes.

#### 2.4.2 Differential expression and ROC curve analysis

Differential expression and receiver operating characteristic (ROC) curve analyses were performed using the standardized gene expression matrix. Samples were first divided into two groups based on grouping information embedded within the sample names. Expression data for the key genes selected by LASSO regression were then extracted. A two-sided t-test was applied to analyze expression differences between the control and experimental groups. To assess the expression-based discriminatory ability of each candidate gene, ROC curves were generated and the area under the curve (AUC) was computed. These analyses were used for exploratory candidate evaluation rather than for clinical diagnostic application. Additionally, box plots and density plots were generated to visualize the expression differences and distributions of each gene across the two groups.

#### 2.4.3 Exploratory ANN-Based candidate prioritization

An exploratory ANN-based model was constructed using the five candidate genes as input variables to provide supportive candidate-prioritization evidence. Samples were encoded for binary classification, with GC samples assigned as 1 and NC samples assigned as 0. The model contained one hidden layer with five neurons and a single output node representing the predicted probability of the GC class. ROC analysis was used to evaluate expression-based separability in the training and validation sets, while individual gene ROC curves were analyzed separately as complementary single-gene discriminatory analyses. Given the limited sample size, the ANN analysis was interpreted strictly as an exploratory screening step rather than as a clinically validated prediction tool.

#### 2.4.4 External validation of candidate gene expression in TCGA-STAD

To further evaluate the robustness of the prioritized candidate genes, external validation was performed using TCGA-STAD data. Expression differences between gastric tumor and normal tissues were analyzed for the five candidate genes, and ROC curves were generated to assess expression-based discriminatory ability in the external dataset. P values for multi-gene expression comparisons were adjusted using the Benjamini-Hochberg method. These external analyses were used to support candidate-gene robustness and were not intended for clinical diagnostic application.

### 2.5 Immune infiltration and consensus clustering analysis

#### 2.5.1 Immune infiltration analysis

The gene expression matrix was subjected to quantile normalization using the limma package in R. Subsequently, CIBERSORT was employed to deconvolute the expression matrix and estimate the relative abundance of immune cell subsets for each sample. Downstream analyses were performed based on these estimated immune cell proportions. Pairwise correlations among different immune cell subsets were calculated using Pearson’s correlation coefficient and visualized in a correlation heatmap. Box plots were generated using ggplot2 and ggpubr packages to compare immune cell infiltration levels between different clinical groups, with Student’s t-tests applied for comparison. For specific cell types, the Wilcoxon rank-sum test was utilized. A filtered heatmap of immune cell proportions was created using pheatmap. Additionally, stacked bar plots were generated to depict the overall immune cell composition for each sample, characterizing the comprehensive immune infiltration landscape.

#### 2.5.2 Consensus clustering analysis

To identify robust molecular subtypes, unsupervised consensus clustering analysis was performed based on the normalized gene expression data. The preprocessed data was imported into R and converted into a numeric matrix. Using the ConsensusClusterPlus package, clustering was executed with the k-means algorithm and Euclidean distance as the similarity metric. Key parameters were set as follows: maximum cluster number (maxK) = 9, number of resampling repetitions (reps) = 1000, proportion of items sampled in each iteration (pItem) = 0.8, and proportion of features sampled (pFeature) = 1 (i.e., all features retained). For each potential cluster number (k), the stability of the clustering solution was evaluated by examining the consensus matrix, the cumulative distribution function (CDF) curves, and the cluster consistency and item consistency indices generated by the calcICL function. Based on this comprehensive assessment, the optimal number of clusters was determined to be k = 2. The consensus subtype assignment for each sample was then extracted for subsequent analyses.

### 2.6 Single-cell and spatial transcriptomic data analysis

Single-cell expression matrix data (GSE163558) were preprocessed using R packages including “Seurat”, “harmony”, “clustree,” “ggplot2,” “patchwork,” and “dplyr.” Data were normalized using the “LogNormalize” method, and highly variable genes were identified via the “vst” selection method. Linear dimensionality reduction was performed using principal component analysis (PCA). The first 10 principal components (PC1:10), determined by examining the elbow plot, were selected for downstream analysis. Subsequently, non-linear dimensionality reduction was carried out using the Uniform Manifold Approximation and Projection (UMAP) algorithm. Cell neighborhoods were identified based on the selected principal components, and cell clustering was performed at a resolution of 0.5. The results were visualized on UMAP plots with cluster annotations. Following preprocessing, cell types were manually annotated and validated. Marker genes for each cluster were identified using the “FindAllMarkers” function. Automated cell type annotation was performed with SingleR, which mapped the 17 Seurat clusters to 10 major cell types (e.g., Epithelial cells, NK cells, T cells). Cell type distribution was visualized using “DimPlot”. Finally, pseudotime trajectory analysis was conducted on the single-cell transcriptomic data using the Monocle package.

### 2.7 Molecular docking

The three-dimensional structure files of the target proteins corresponding to the key genes were retrieved in PDB format from the RCSB Protein Data Bank (https://www.rcsb.org/). The molecular structure file of BaP was obtained from PubChem. Molecular docking analysis was performed using the CB-Dock2 web server (https://cadd.labshare.cn/cb-dock2/). The binding affinity (kcal/mol) was used to evaluate the predicted interaction strength between the ligand (BaP) and the receptor (target protein). A binding affinity lower than −3 kcal/mol suggests spontaneous binding, while a value lower than −5 kcal/mol indicates a favorable predicted docking pose. A two-dimensional ligand-residue interaction diagram was generated to visualize the predicted local binding environment. Docking results were interpreted as *in silico* structural predictions rather than direct experimental evidence of physical binding.

### 2.8 Immunohistochemistry (IHC) staining

Immunohistochemistry images utilized in this study were sourced from the Human Protein Atlas database (https://www.proteinatlas.org/). Staining results for both tumor tissues and their corresponding normal tissues were retrieved. It should be noted that due to the absence of IHC data for Alcohol Dehydrogenase 7 (ADH7) in the database, this molecule was not included in the related comparative analysis.

### 2.9 Survival analysis of key target genes

Survival analysis for the five key target genes (ADH7, CA9, COL4A1, ENO1, GPT) was performed using the Kaplan-Meier Plotter online tool (https://kmplot.com/) to evaluate the correlation between the expression of these core targets and the overall survival of gastric cancer patients.

### 2.10 Acquisition and differential analysis of ENO1 gene expression data

Expression data for the ENO1 gene across multiple tumor types and paired adjacent normal tissues were obtained from The Cancer Genome Atlas (TCGA) database via the TIMER2.0 online analysis platform (https://compbio.cn/timer2/). The distribution of gene expression levels was visualized using box plots. Differential expression analysis was conducted based on TCGA RNA-Seq raw count data utilizing the edgeR algorithm. Statistical significance is denoted by asterisks in the figures (*P < 0.05, **P < 0.01, ***P < 0.001).

### 2.11 Analysis of ENO1 expression features in gastric cancer tissues

Expression characteristics of the ENO1 gene in gastric cancer were analyzed using the GEPIA2 platform (http://gepia2.cancer-pku.cn/). Transcriptomic data from gastric cancer tissues and normal gastric mucosa were extracted. The expression distribution of ENO1 was visualized via box plots. Statistical significance of expression differences, as analyzed by the platform’s built-in methods, is indicated by asterisks in figures (P < 0.05, P < 0.01, P < 0.001).

### 2.12 Cell culture

The human gastric cancer cell lines MGC803 and HGC-27 were used in this study. Cells were cultured in RPMI-1640 medium (Gibco, United States) supplemented with 10% fetal bovine serum (FBS, Gibco, United States) and 1% penicillin-streptomycin solution (Beyotime Biotechnology, China). All cells were maintained in a sterile, humidified incubator at 37 °C with 5% CO_2_.

### 2.13 Wound healing (scratch) assay

Cell migration ability was assessed using a mechanical scratch assay. Cells were seeded into 6-well plates at a density of 5 × 10^5^ cells/well and allowed to adhere for 24 h. A uniform scratch was then created across the confluent monolayer using a sterile 200 µL pipette tip, guided by pre-marked perpendicular lines drawn with a sterile marker. The wells were gently washed three times with sterile PBS to remove cell debris and replenished with serum-free medium (or low-serum medium containing ≤2% FBS). Cells were returned to the incubator (37 °C, 5% CO_2_). Migration into the scratch area was dynamically monitored at predefined positions at 0, 24, and 48 h using an inverted phase-contrast microscope. Images were captured for quantitative analysis.

### 2.14 Transwell migration assay

Cell migratory ability was evaluated using a transwell migration assay without Matrigel coating. Briefly, cells were seeded into the upper chamber of a transwell insert at a density of 3 × 10^4^ cells per well in serum-free medium. The lower chamber was filled with medium supplemented with 10% FBS as a chemoattractant. After incubation at 37 °C in a humidified atmosphere containing 5% CO_2_ for 48 h, non-migrated cells on the upper surface of the membrane were gently removed with a cotton swab. Cells that had migrated to the lower surface of the membrane were fixed and stained with 0.1% crystal violet for 30 min, then imaged and counted under a microscope.

### 2.15 Colony formation assay

Cells were seeded into 6-well plates at a density of 1,500 cells per well and cultured continuously for 2 weeks in the presence of either DMSO (vehicle control) or various concentrations of BaP (0, 0.1, 0.5, and 1.0 μM). After the incubation period, colonies were fixed and stained with 0.1% crystal violet (Sichuan Superior Chemicals & Products Co., Ltd.) for 30 min, followed by two washes with phosphate-buffered saline (PBS). Colonies with a diameter larger than 1 mm were manually counted.

### 2.16 EdU assay for cell proliferation

Cells in the logarithmic growth phase were seeded into 6-well plates. After 24 h of adhesion, the culture medium was replaced with complete medium containing 10 μM EdU (5-ethynyl-2′-deoxyuridine), and cells were incubated for an additional 2 h. The medium was then aspirated, and cells were fixed with 4% paraformaldehyde for 15 min, followed by washing and permeabilization. A Click reaction cocktail was added to detect the incorporated EdU, and the plate was incubated in the dark for 30 min. After washing, cell nuclei were stained with Hoechst 33342 for 10 min. Images were captured under a fluorescence microscope. The proportion of EdU-positive cells was calculated from randomly selected fields and analyzed using ImageJ software.

### 2.17 CCK-8 cell proliferation assay

Cells in the logarithmic growth phase were harvested, counted, and seeded into 96-well plates. Experimental design included blank control (medium only), negative control (vehicle-treated), and various treatment groups, with 3-6 replicate wells per group. After the designated treatment period, 10 μL of CCK-8 reagent was added to each well, and the plate was incubated in the dark for 1 h. The absorbance (optical density, OD) of each well was measured at 450 nm using a microplate reader. The average OD value for each group was calculated after subtracting the blank control value and used to compare relative cell proliferation activity.

### 2.18 siRNA construction and cell transfection

Cells were evenly seeded into 6-well plates. When cell density reached approximately 40%, transfection was initiated. siRNA targeting ENO1 was transfected into MGC803 and HGC-27 cells using Lipofectamine 2000 according to the manufacturer’s protocol. After 4–6 h of incubation, the medium was replaced with fresh complete medium, and cells were cultured for an additional 24–48 h. Cells were then harvested for validation of knockdown efficiency by qRT-PCR and Western blotting. Following successful validation, subsequent experiments were performed. The control and ENO1 gene knockdown sequences were synthesized by GenePharma (Shanghai, China). All siRNA sequences are listed in Supplementary Table S1.

### 2.19 Western blot analysis

Treated cells were lysed on ice for 30 min using cold RIPA lysis buffer supplemented with protease inhibitors, including PMSF. Lysates were centrifuged at high speed, and the supernatant was collected. Protein concentration was determined using a BCA protein assay kit. Equal amounts of protein were separated by SDS-PAGE and subsequently transferred onto PVDF membranes via wet transfer. After transfer, membranes were blocked with rapid blocking buffer for 10 min at room temperature, followed by incubation with primary antibodies overnight at 4 °C. Membranes were washed with TBST and then incubated with HRP-conjugated secondary antibodies for 1 h at room temperature. After thorough washing, protein bands were visualized using a hypersensitive chemiluminescence detection reagent and imaged with a digital imaging system. Band intensity was quantified using ImageJ software and normalized to the corresponding loading control. Details of all antibodies used are provided in Supplementary Table S2.

### 2.20 Quantitative real-time PCR (qRT-PCR)

Total RNA was extracted from treated cells using TRIzol reagent. cDNA was synthesized from RNA using a PrimeScript RT Reagent Kit. qRT-PCR was performed using QuantiNova SYBR Green PCR Master Mix on a real-time PCR detection system. β-actin was used as the endogenous reference gene. All primer sequences used for qRT-PCR are listed in Supplementary Table S3. Each sample was run in triplicate. The relative expression level of the target gene was calculated using the 2^−ΔΔCT^ method.

### 2.21 Statistical analysis

All *in vitro* experiments were performed with at least three independent biological replicates, with technical replicates included where appropriate. Data visualization and quantification were conducted using Fiji ImageJ and GraphPad Prism 8.0.1. For bioinformatics analyses involving multiple testing, P values were adjusted using the Benjamini-Hochberg method unless otherwise specified. For *in vitro* experiments, comparisons between two groups were performed using Students t-test, whereas comparisons among three or more groups were performed using one-way ANOVA followed by *post hoc* multiple-comparison testing. A P value or adjusted P value less than 0.05 was considered statistically significant. For public transcriptomic and single-cell datasets, sample sizes were determined by the availability of eligible samples in the original datasets; therefore, formal *a priori* power analysis was not applicable. For *in vitro* assays, replicate numbers were selected according to common practice in exploratory cell-based studies and previous related reports.

## 3 Result

### 3.1 Identification of Benzo [a]pyrene–gastric cancer interaction targets

**FIGURE 1:**
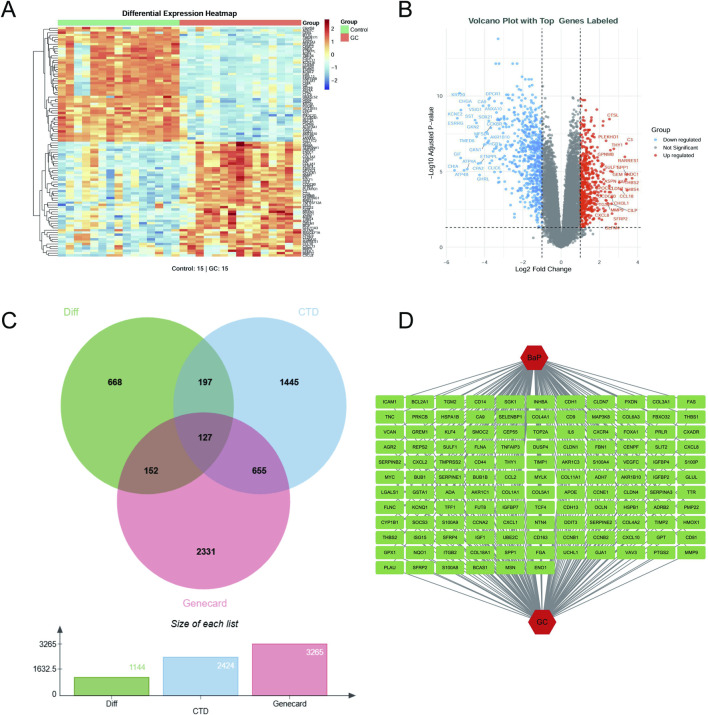
Identification of Benzo [a]pyrene–gastric cancer interaction targets. **(A)** Heatmap showing differentially expressed genes (DEGs) between gastric tumor and adjacent normal tissues from the GSE118916 dataset. **(B)** Volcano plot displaying the distribution of DEGs; red dots represent significantly upregulated genes, blue dots represent significantly downregulated genes, and gray dots indicate non-significant genes. **(C)** Venn diagram illustrating the overlap among CTD-derived BaP-associated targets, GeneCards-derived gastric cancer-related genes, and GSE118916-derived DEGs. The three-way intersection contained 127 genes, which were defined as BaP-gastric cancer interaction targets for downstream analyses. **(D)** Protein–protein interaction (PPI) network of the intersecting targets constructed using the STRING database.

To systematically identify potential molecular targets linking Benzo [a]pyrene (BaP) exposure to gastric cancer, we first retrieved the chemical structure and SMILES sequence of BaP from the PubChem database. Potential BaP-binding targets were predicted using the Comparative Toxicogenomics Database (CTD) with an interaction count threshold >5. Concurrently, gastric cancer-related targets were collected from the GeneCards database (Relevance score >10). To enhance clinical relevance, we analyzed the GSE118916 dataset from the GEO database to identify significantly differentially expressed genes (DEGs) between gastric tumor and adjacent normal tissues, visualizing the results via heatmap and volcano plot (Figures 1A,B). The intersection of these three datasets—predicted BaP targets, gastric cancer-related targets, and gastric cancer DEGs—defined a core set of “BaP–gastric cancer interaction targets” (Figure 1C). Subsequently, a protein–protein interaction (PPI) network was constructed using the STRING database to elucidate the functional relationships among these core targets, providing a foundation for subsequent analyses (Figure 1D).

### 3.2 Functional enrichment analysis of the intersecting targets

**FIGURE 2:**
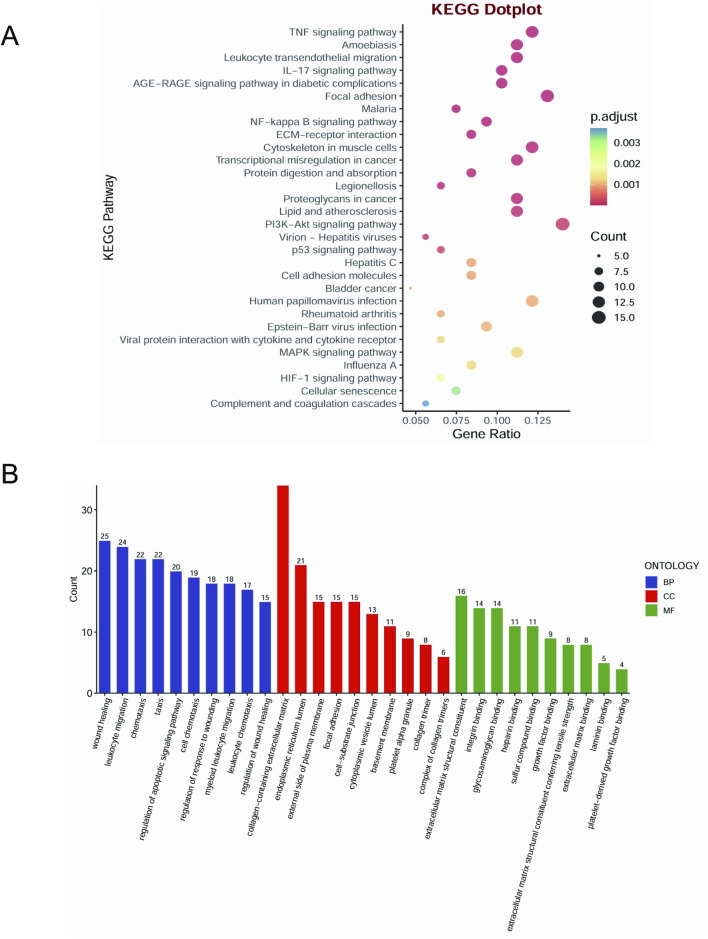
Functional enrichment analysis of BaP–gastric cancer intersecting targets. **(A)** Kyoto Encyclopedia of Genes and Genomes (KEGG) pathway enrichment analysis showing significantly enriched signaling pathways associated with the intersecting targets. **(B)** Gene Ontology (GO) enrichment analysis results, including Biological Process (BP), Cellular Component (CC), and Molecular Function (MF) categories.

To elucidate the biological functions and core signaling pathways associated with the BaP-gastric cancer intersecting targets identified from the PPI network, Gene Ontology (GO) annotation and Kyoto Encyclopedia of Genes and Genomes (KEGG) pathway enrichment analyses were performed (Figures 2A,B). KEGG pathway analysis revealed that these targets were significantly enriched in key pathways closely related to tumorigenesis and progression, including focal adhesion, PI3K-Akt signaling, p53 signaling, transcriptional misregulation in cancer, TNF signaling, and HIF-1 signaling. GO functional annotation further characterized the targets across three categories. For Biological Process (BP), significantly enriched terms included leukocyte migration, regulation of wound healing, and regulation of apoptotic signaling pathway. Regarding Cellular Component (CC), targets were primarily localized to the collagen-containing extracellular matrix, cell-substrate junction, and focal adhesion. In terms of Molecular Function (MF), the dominant functions included extracellular matrix structural constituent, laminin binding, and extracellular matrix binding. These enrichment results provided a functional background for the intersecting target set and supported subsequent candidate prioritization.

### 3.3 Exploratory prioritization of BaP-Associated candidate genes

**FIGURE 3:**
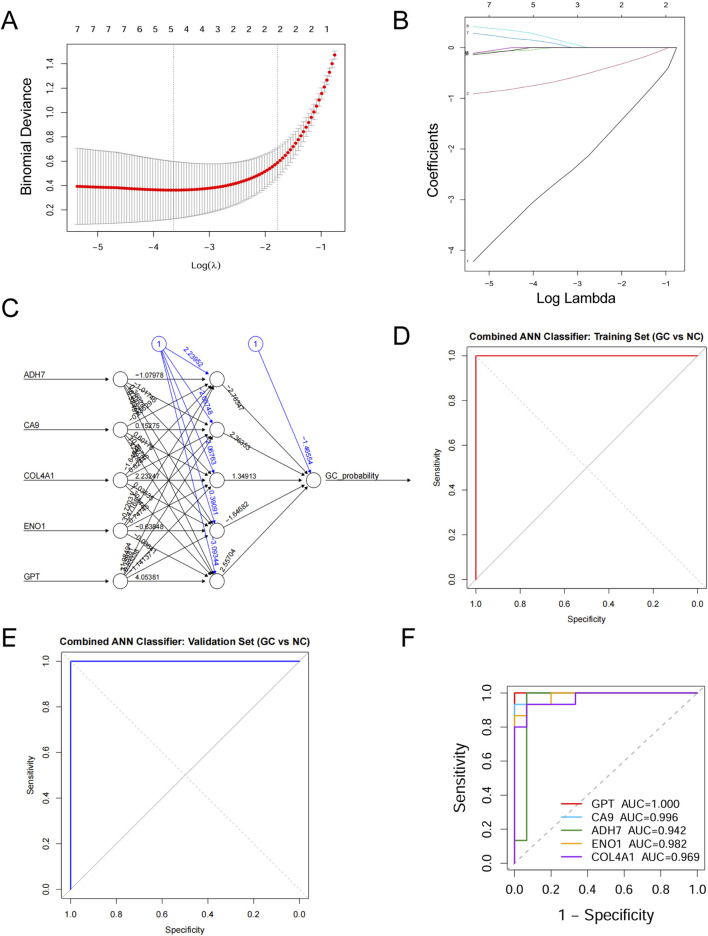
Exploratory prioritization of key benzo [a]pyrene-associated candidate genes. **(A, B)** Least Absolute Shrinkage and Selection Operator (LASSO) regression analysis identifying five candidate genes: ADH7, CA9, COL4A1, ENO1, and GPT. **(A)** Cross-validation curve for optimal lambda selection. **(B)** LASSO coefficient profiles of candidate genes. **(C)** Architecture of the exploratory ANN-based model used as a supportive candidate-prioritization step. The model uses the five selected genes as input variables, one hidden layer containing five neurons, and a single output node representing expression-based separability of GC and NC samples. **(D, E)** Receiver Operating Characteristic (ROC) curves evaluating expression-based separability of the combined five-gene exploratory ANN model in the training set **(D)** and validation set **(E)**. **(F)** Individual ROC curves generated separately for each candidate gene as complementary single-gene discriminatory analyses.

To prioritize candidate genes from the BaP-gastric cancer intersecting targets, we first applied Least Absolute Shrinkage and Selection Operator (LASSO) regression analysis. This exploratory feature-screening approach yielded five candidate genes: ADH7, CA9, COL4A1, ENO1, and GPT (Figures 3A,B). To further support candidate prioritization, an exploratory ANN-based model was constructed using their expression profiles (Figure 3C). ROC curves in the training and validation sets were used to evaluate expression-based separability of the five-gene model in the analyzed dataset (Figures 3D,E). Separately, individual ROC curves were generated for each candidate gene as complementary single-gene analyses and were not intended to represent the performance of the combined ANN model (Figure 3F). Because of the limited sample size, these machine-learning analyses were interpreted as exploratory candidate-screening procedures rather than as clinically validated modeling.

### 3.4 Expression and survival analysis of key target genes in gastric cancer

**FIGURE 4:**
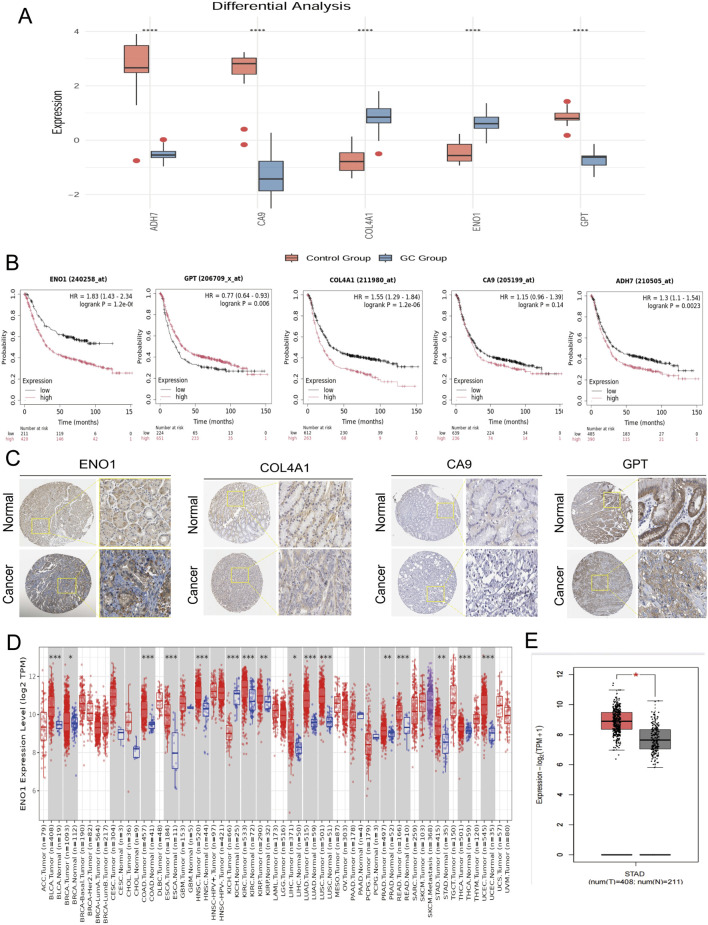
Expression and clinical-context support for prioritized candidate genes in gastric cancer. **(A)** Differential analysis of mRNA expression levels of the five prioritized candidate genes between gastric tumor and normal tissues. **(B)** Kaplan–Meier survival analysis showing the association between ENO1 expression and patient prognosis; high ENO1 expression was significantly associated with poor overall survival. **(C)** Immunohistochemical staining images from the Human Protein Atlas (HPA) database confirming elevated protein levels of key targets in gastric cancer tissues. **(D)** Pan-cancer analysis of ENO1 expression across multiple cancer types. **(E)** ENO1 mRNA expression comparison between gastric cancer tissues and normal gastric mucosa.

To evaluate the expression patterns and clinical-context support for the prioritized candidate genes, we performed a series of public-database analyses. Compared with normal tissues, ENO1 mRNA expression was significantly elevated in gastric tumor tissues (Figure 4A). Survival analysis revealed that high ENO1 expression was significantly associated with poorer patient prognosis (Figure 4B). Furthermore, immunohistochemical data from the Human Protein Atlas (HPA) database supported increased ENO1 protein expression in gastric cancer samples (Figure 4C).

Integrating evidence from LASSO selection, exploratory ANN-based prioritization, external expression validation, survival association, and subsequent multi-omics characterization, ENO1 was selected for downstream validation because it showed the most convergent support across multiple analytical layers. Pan-cancer analysis further demonstrated that ENO1 expression is elevated across multiple cancer types, including gastric cancer (Figure 4D). Focusing specifically on gastric cancer, ENO1 expression was significantly higher in tumor tissues compared with normal gastric mucosa (Figure 4E). External TCGA-STAD validation further supported differential expression and moderate expression-based discriminatory ability of ENO1 and other prioritized candidates (Supplementary Figure S1). These findings supported ENO1 as the primary candidate for experimental validation, whereas the other candidate genes were retained as part of the exploratory screening set.

### 3.5 Single-cell contextualization of ENO1 in gastric cancer

**FIGURE 5:**
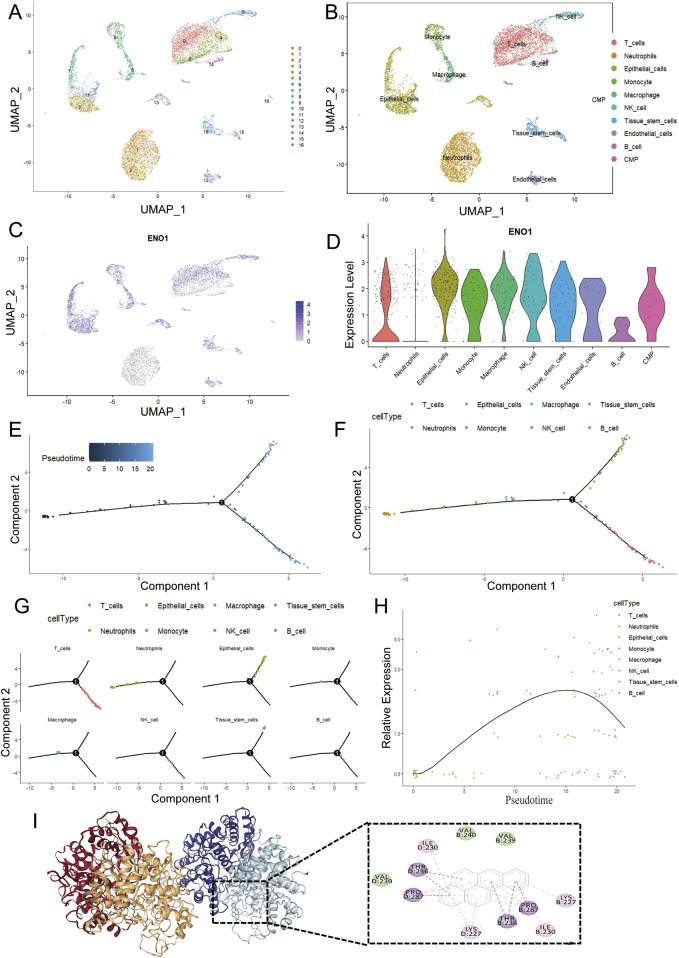
Single-cell contextualization and molecular docking analysis of ENO1. **(A)** UMAP visualization of cell clusters identified from the GSE163558 single-cell RNA sequencing dataset. **(B)** Annotation of 17 cell clusters into 10 major cell types using SingleR. **(C, D)** Expression distribution of ENO1 across different cell types. **(E)** Shared global pseudotime trajectory colored by pseudotime value. **(F)** Distribution of annotated cell types along the same shared trajectory. **(G)** Faceted visualization showing the position of each major cell type on the shared pseudotime trajectory. **(H)** ENO1 expression dynamics along the shared global pseudotime trajectory shown in panels E–G. **(I)** Molecular docking analysis of BaP with ENO1. The left panel shows the predicted overall docking pose, and the right panel shows a two-dimensional residue-level interaction diagram. The predicted binding environment involved residues including VAL239/VAL240, LYS227, ILE230, THR236, and PRO287, with van der Waals, Pi-alkyl, and Pi-sigma interactions. The docking result is presented as an *in silico* structural prediction rather than direct experimental evidence of physical binding.

To deconvolute cellular heterogeneity within the gastric cancer microenvironment and characterize the cellular context of ENO1 expression, we analyzed the single-cell dataset GSE163558. Using SingleR annotation, the 17 identified cell clusters were classified into 10 major cell types (Figures 5A,B). Expression analysis revealed that ENO1 was highly expressed in epithelial cells and specific immune cell populations, particularly macrophages and T cells (Figures 5C,D), suggesting that ENO1 may be associated with both tumor-cell and microenvironment-related cellular states. Pseudotime analysis was performed to explore the shared global cell-state ordering of the analyzed single-cell population. The inferred trajectory was displayed according to pseudotime values and then mapped by annotated cell types, showing that epithelial cells, T cells, macrophages, and other cell populations occupied distinct regions along the shared trajectory (Figures 5E–G). ENO1 expression dynamics were then examined along this shared global pseudotime trajectory, where ENO1 showed an increasing trend toward later pseudotime states (Figure 5H).

### 3.6 Molecular docking analysis

To explore a possible structural relationship between BaP and ENO1 at the protein level, molecular docking simulations were conducted. The predicted BaP-ENO1 docking pose showed a binding energy lower than −5 kcal/mol, indicating a favorable *in silico* docking result (Figure 5I). A two-dimensional interaction diagram further indicated that BaP was surrounded by residues including VAL239/VAL240, LYS227, ILE230, THR236, and PRO287, with predicted van der Waals, Pi-alkyl, and Pi-sigma interactions. These residue-level interactions are consistent with the hydrophobic polycyclic aromatic structure of BaP. However, this docking analysis should be interpreted as an *in silico* structural hypothesis rather than direct experimental evidence of physical binding or as an explanation for BaP-induced ENO1 transcriptional upregulation.

### 3.7 BaP promotes the proliferation and migration of gastric cancer cells

**FIGURE 6:**
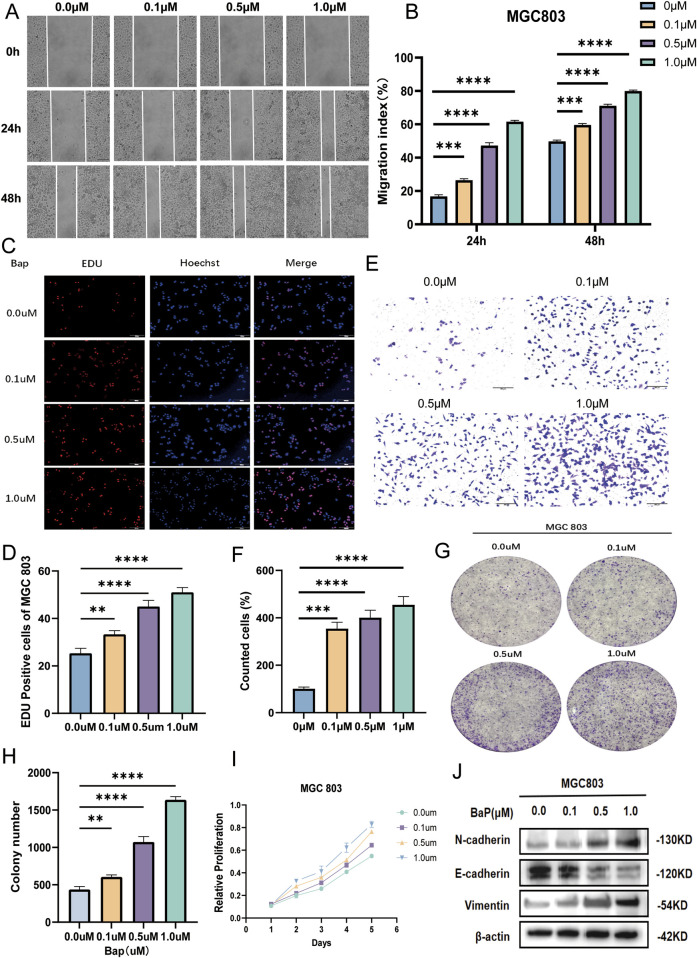
BaP promotes the proliferation and migration of gastric cancer cells. MGC803 cells were treated with BaP at 0, 0.1, 0.5, and 1.0 μM for 48 h **(A, B)** Wound healing assay demonstrating concentration-dependent enhancement of cell migration by BaP treatment. **(A)** Representative images at 0 and 48 h **(B)** Quantification of wound closure rate. **(C, D)** EdU incorporation assay showing increased cell proliferation upon BaP treatment. **(C)** Representative fluorescence images. **(D)** Quantification of EdU-positive cells. **(E, F)** Transwell migration assay indicating enhanced invasive capacity with increasing BaP concentrations. **(E)** Representative images. **(F)** Quantification of migrated cells. **(G, H)** Colony formation assay demonstrating BaP-induced enhancement of clonogenic ability **(G)** Representative images. **(H)** Quantification of colony numbers. **(I)** CCK-8 assay confirming the proliferative effect of BaP. **(J)** Western blot analysis of EMT markers showing upregulation of N-cadherin and Vimentin and downregulation of E-cadherin following BaP treatment. *P < 0.05, **P < 0.01, ***P < 0.001, ****P < 0.0001.

To investigate the functional impact of BaP on gastric cancer cells, we established an *in vitro* model using the human gastric cancer cell line MGC803. Based on prior literature (Wei et al., 2016) and preliminary experiments, cells were treated with BaP at concentrations of 0, 0.1, 0.5, and 1.0 μM for 48 h. Functional responses were assessed using wound healing, Transwell migration, EdU incorporation, colony formation, and CCK-8 assays. Wound healing assays revealed a significant, concentration-dependent increase in cell migration after 48 h of BaP treatment (Figures 6A,B). Similarly, Transwell assays showed a progressive increase in the number of invading cells with rising BaP concentrations (Figures 6E,F). EdU assays demonstrated that BaP treatment markedly enhanced cell proliferation (Figures 6C,D), a finding corroborated by colony formation and CCK-8 assays (Figures 6G–I). Western blot analysis further indicated that BaP upregulated the expression of mesenchymal markers (N-cadherin, Vimentin) while downregulating the epithelial marker E-cadherin (Figure 6J), suggesting its potential to induce an epithelial-mesenchymal transition (EMT) phenotype conducive to metastasis.

Collectively, these *in vitro* experiments demonstrate that BaP enhances the malignant behaviors of MGC803 cells—including proliferation, migration, and invasion—in a concentration-dependent manner (0–1.0 μM).

### 3.8 ENO1 contributes to BaP-Associated proliferation and migration in gastric cancer cells

**FIGURE 7:**
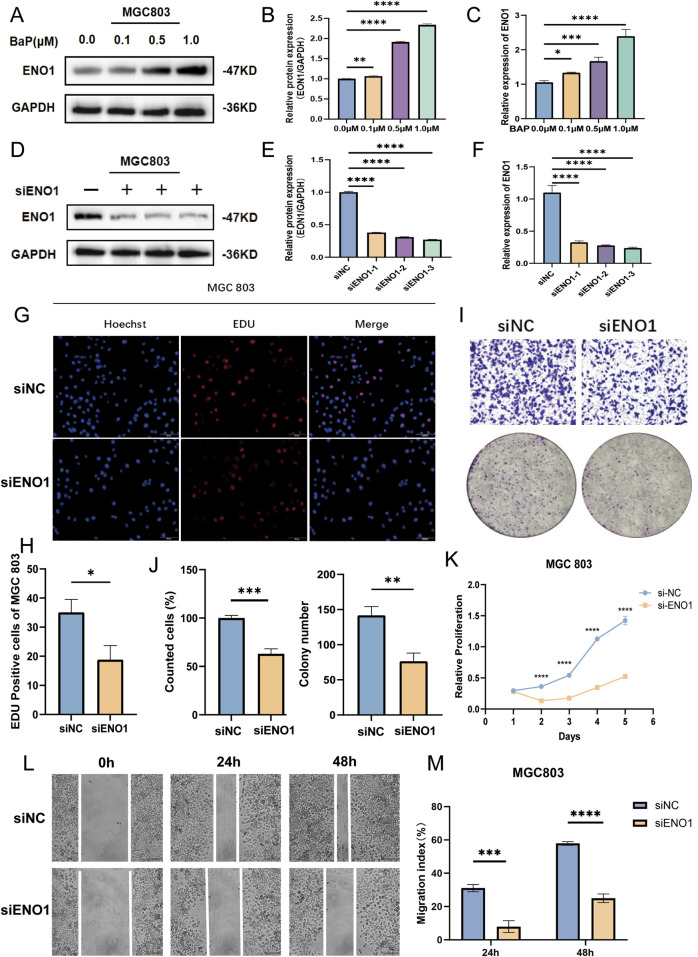
ENO1 knockdown suppresses gastric cancer cell proliferation and migration. **(A–C)** BaP treatment upregulates ENO1 expression in a dose-dependent manner. **(A)** Western blot analysis of ENO1 protein levels. **(B)** Quantification of ENO1 protein expression. **(C)** qRT-PCR analysis of ENO1 mRNA levels. **(D–F)** Verification of siRNA-mediated ENO1 knockdown efficiency. **(D)** Western blot analysis. **(E)** Quantification of knockdown efficiency at protein level. **(F)** qRT-PCR validation of knockdown efficiency at mRNA level. **(G, H)** EdU assay showing reduced proliferation upon ENO1 knockdown. **(I, J)** Colony formation assay demonstrating decreased clonogenic ability after ENO1 knockdown. **(K)** CCK-8 assay confirming suppressed proliferation **(L, M)** Transwell and wound healing assays indicating attenuated migration following ENO1 knockdown. *P < 0.05, **P < 0.01, ***P < 0.001, ****P < 0.0001.

Given our prior identification of ENO1 as a candidate target in the BaP-associated gastric cancer network, we first evaluated its regulation after BaP exposure. Western blot and qRT-PCR analyses showed that both ENO1 protein and mRNA levels increased in a dose-dependent manner following BaP treatment (Figures 7A–C).

To directly assess the functional role of ENO1 in gastric cancer progression, we performed siRNA-mediated knockdown in MGC803 cells, with knockdown efficiency verified by Western blot and qRT-PCR (Figures 7D–F). Functional assays demonstrated that knockdown of ENO1 significantly suppressed gastric cancer cell proliferation, as evidenced by EdU incorporation, colony formation, and CCK-8 assays, and attenuated cell migration, as shown by Transwell and wound healing assays (Figures 7G–M).

**FIGURE 8:**
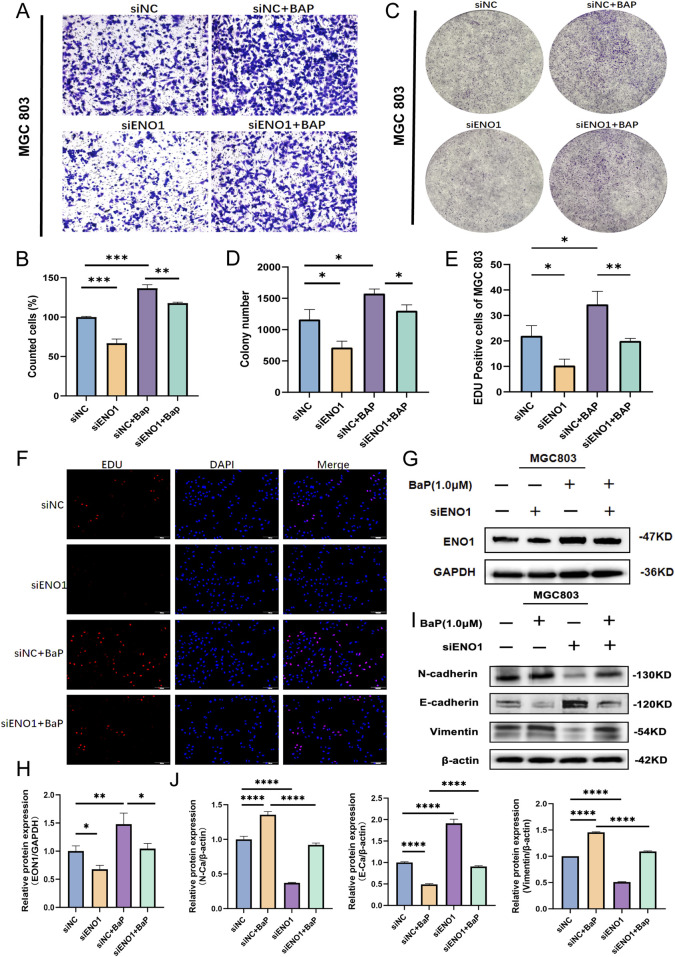
ENO1 knockdown attenuates BaP-associated malignant phenotypes in gastric cancer cells. Knockdown-based attenuation experiments were performed in ENO1-knockdown MGC803 cells treated with 1.0 μM BaP. **(A, B)** Transwell assay demonstrating that ENO1 knockdown attenuated BaP-enhanced migration. **(A)** Representative images. **(B)** Quantification of migrated cells. **(C, D)** Colony formation assay showing attenuation of BaP-induced clonogenic enhancement by ENO1 knockdown. **(C)** Representative images. **(D)** Quantification of colony numbers. **(E, F)** EdU assay indicating that ENO1 knockdown reduced BaP-promoted proliferation. **(E)** Representative EdU images. **(F)** Quantification of EdU-positive cells. **(G, H)** Western blot analysis showing partial restoration of ENO1 protein levels by BaP treatment in knockdown cells. **(I, J)** Western blot analysis of EMT-related markers showing that ENO1 knockdown attenuated the BaP-associated decrease in E-cadherin and the increase in N-cadherin and vimentin. The siNC group served as the negative siRNA control, siNC + BaP served as the positive BaP-response control, and siENO1 + BaP was used to assess whether ENO1 knockdown attenuated BaP-associated phenotypes. *P < 0.05, **P < 0.01, ***P < 0.001, ****P < 0.0001.

Knockdown-based attenuation experiments were conducted to evaluate the relationship between BaP-associated phenotypes and ENO1. The experiments included siNC, siNC + BaP, siENO1, and siENO1 + BaP groups. The siNC group served as the negative siRNA control under vehicle-treated conditions, whereas the siNC + BaP group served as the positive BaP-response control. The siENO1 group was used to assess the effect of ENO1 knockdown alone, and the siENO1 + BaP group was used to determine whether ENO1 knockdown attenuated BaP-associated cellular phenotypes. In ENO1-knockdown cells, the pro-migratory effect induced by 1.0 μM BaP was attenuated in Transwell assays (Figures 8A,B). Similarly, BaP-enhanced proliferation was reduced, as shown by colony formation and EdU assays (Figures 8C–F). Western blot analysis indicated that BaP treatment partially restored ENO1 protein levels in knockdown cells (Figures 8G,H). Furthermore, ENO1 knockdown attenuated the BaP-associated decrease in E-cadherin and the increase in N-cadherin and Vimentin (Figures 8I,J).

Taken together, these *in vitro* findings support that ENO1 contributes, at least in part, to BaP-associated proliferation, migration, and EMT-like changes in gastric cancer cells.

To further examine whether the BaP-ENO1 association was restricted to MGC803 cells, additional validation was performed in HGC-27 gastric cancer cells. In a four-group qRT-PCR experiment, BaP increased ENO1 mRNA expression under siNC conditions, whereas siENO1 reduced basal ENO1 expression and attenuated BaP-associated ENO1 induction. In parallel, BaP enhanced HGC-27 cell migration and clonogenic capacity across the concentration gradient of 0, 0.1, 0.5, and 1.0 μM, and ENO1 knockdown reduced clonogenic capacity in HGC-27 cells (Supplementary Figure S2). These additional data support that BaP-associated ENO1 induction and ENO1-related migration and proliferation-associated phenotypes are not restricted to a single gastric cancer cell line, although broader validation remains necessary.

## 4 Discussion

Polycyclic aromatic hydrocarbons (PAHs), a class of persistent organic pollutants ubiquitous in the environment, are primarily generated from the incomplete combustion of carbon-containing materials such as fossil fuels (Shen et al., 2013). Among them, Benzo [a]pyrene (BaP), recognized as a key indicator of integrated PAH exposure due to its potent carcinogenicity and widespread environmental presence, is characterized by exceptional chemical stability and strong hydrophobicity. Primary sources of human exposure include tobacco smoke, vehicle emissions, coal burning, and the consumption of fried, grilled, roasted, or smoked foods (Kim et al., 2013; Porwisiak et al., 2023). Prospective epidemiological studies have established a significant positive correlation between internal BaP exposure levels and gastric cancer risk in humans (Agudo et al., 2012; Zhao et al., 2025). However, the comprehensive molecular network underlying its carcinogenic effects remains incompletely elucidated. Existing mechanistic investigations, such as those focusing on the AhR signaling pathway, have primarily revealed specific routes of BaP action (Wei et al., 2016). Given the complexity of BaP’s toxic effects, a systematic approach to identify its key molecular targets is crucial. Therefore, to efficiently uncover these critical targets, this study integrated network toxicology and computational biology strategies, aiming to explore the potential key targets of BaP in gastric cancer through systematic screening. In addition, based on concentration gradients reported in the existing literature, we established corresponding *in vitro* cell models to validate the role of potential key targets in the BaP–gastric cancer axis (Wei et al., 2016).

Network toxicology provides a useful framework for identifying candidate molecular nodes within complex biological networks affected by environmental carcinogens. By integrating network toxicology methods, we identified targets potentially related to BaP and constructed a PPI network comprising 127 proteins. Using LASSO regression analysis and exploratory ANN-based prioritization, we refined this network to five candidate genes: ADH7, CA9, COL4A1, ENO1, and GPT. Because the ANN analysis was performed on a limited dataset, it was used only as a supportive screening step rather than as a clinically validated prediction tool. Across the subsequent analytical layers, ENO1 showed the most convergent support, including expression elevation, survival association, single-cell contextualization, docking-based structural prediction, BaP-responsive expression changes, and attenuation of BaP-associated phenotypes after ENO1 knockdown. Thus, the revised interpretation focuses on ENO1 as the primary experimentally followed candidate rather than presenting multiple co-candidates.

ENO1, a key glycolytic enzyme (Muller et al., 2012), is a multifunctional molecule implicated in gastric cancer biology. Previous studies have reported that ENO1 can be regulated at multiple levels, including post-translational modification such as SENP1-mediated deSUMOylation that enhances ENO1 stability (Fang et al., 2025), and post-transcriptional regulation such as miR-22-3p-mediated repression (Qiao et al., 2023). ENO1 has also been linked to downstream oncogenic signaling such as AKT activation (Sun et al., 2019; Shu et al., 2025). In the present study, BaP increased ENO1 mRNA and protein expression in gastric cancer cells, and the additional HGC-27 qRT-PCR experiment further supported BaP-associated ENO1 induction at the transcript level. However, these data do not distinguish whether the observed increase in ENO1 mRNA results from transcriptional activation, altered mRNA stability, or upstream post-transcriptional regulation, nor do they determine whether increased ENO1 protein reflects enhanced translation or protein stabilization. Similarly, although BaP is classically linked to AhR-dependent transcriptional responses, the present study did not test whether AhR is required for ENO1 induction. Therefore, SENP1-mediated protein stabilization, miR-22-3p-related post-transcriptional regulation, AhR-dependent or AhR-independent transcriptional regulation, oxidative stress, hypoxia-associated signaling, and metabolic stress should be regarded as mechanistic hypotheses for future investigation rather than mechanisms demonstrated in the present work.

Beyond ENO1, the other four candidate genes may also reflect biologically meaningful aspects of gastric cancer progression. In particular, COL4A1 showed strong expression-based separability in TCGA-STAD and may be related to extracellular matrix remodeling and tumor microenvironment-associated processes. However, expression-based separability alone was not used as the decisive criterion for final experimental prioritization. Compared with COL4A1, ENO1 showed broader convergence across the analytical and experimental layers used in this study: it was selected by the exploratory screening workflow, was associated with adverse survival, showed elevated expression in gastric cancer and relevant single-cell populations, had a plausible docking-based structural hypothesis with BaP, was induced by BaP at the mRNA/protein level, and its knockdown attenuated BaP-associated malignant phenotypes. By contrast, COL4A1 was not experimentally tested for BaP responsiveness or loss-of-function attenuation in the present study. For this reason, we did not present ENO1 and COL4A1 as co-primary mediators. COL4A1 and the remaining candidates are retained as exploratory candidates that may warrant future validation, whereas ENO1 is emphasized as the primary experimentally followed candidate in the revised manuscript.

The main contribution of this study lies in the systematic prioritization of ENO1 as a candidate mediator linking benzo [a]pyrene (BaP) exposure to gastric cancer progression through an integrative toxicogenomic and multi-omics framework. Rather than establishing a fully resolved mechanistic pathway or clinical application, our findings provide preliminary biological support for the involvement of ENO1 in BaP-associated malignant phenotypes. The elevated expression of ENO1 in tumor epithelial cells and selected immune cell populations suggests possible relevance to both tumor-intrinsic and microenvironment-associated cellular states, but these observations remain hypothesis-generating. Therefore, ENO1 should be considered a candidate mediator and candidate biomarker for further validation, rather than an established therapeutic target.

Several methodological limitations of the present computational workflow should also be acknowledged. First, because the prioritization process integrates curated resources such as CTD, GeneCards, and STRING, the resulting candidate list may be influenced by literature bias and may preferentially retain highly studied or highly connected genes. Second, overlap among databases may introduce redundancy, and the intersection-based strategy used here is more suitable for candidate prioritization than for claiming *de novo* mechanistic discovery. Third, because feature selection was performed on a relatively limited transcriptomic dataset, the stability of gene selection and model performance may be sensitive to sampling variation and parameter settings. Accordingly, the machine-learning analyses should be interpreted as exploratory candidate-screening procedures rather than as clinically validated prediction tools. Fourth, the TCGA-based analyses may be affected by clinical and molecular confounders, including treatment history, tumor stage, mutation status, molecular subtype, tumor purity, and differences in sample processing. Finally, the single-cell analysis was based on the public GSE163558 dataset and may not fully represent the cellular heterogeneity of all gastric cancer subtypes, stages, or BaP exposure backgrounds. Moreover, the shared pseudotime analysis provides an overview of global cell-state ordering but does not replace dedicated lineage-specific trajectory analyses for epithelial cells, macrophages, or T-cell subsets.

Concurrently, the following limitations of the experimental work must be explicitly acknowledged. First, the main conclusions still lack direct validation in vivo animal models and clinical cohorts based on authentic human exposure levels. Second, although BaP increased both ENO1 mRNA and protein levels, the precise upstream molecular events remain unknown. The present study did not distinguish whether BaP-induced ENO1 upregulation occurs through transcriptional activation, altered mRNA stability, translational regulation, or protein stabilization, and the dependence or independence of this response from the AhR pathway remains unresolved. Third, molecular docking provides only an *in silico* structural hypothesis and does not constitute direct experimental evidence of physical BaP-ENO1 binding. Future studies using AhR inhibition or knockdown, ENO1 promoter reporter assays, actinomycin D-based mRNA stability assays, cycloheximide chase assays, SENP1- or miRNA-focused experiments, and direct binding assays such as CETSA, DARTS, SPR, MST, or pull-down analyses will be needed to clarify the upstream mechanism and validate whether BaP physically interacts with ENO1 in living cells. Fourth, although additional validation in HGC-27 cells strengthens the migration- and proliferation-related findings, broader validation across additional gastric cancer cell lines, animal models, and exposure-relevant systems remains necessary. Finally, the knockdown-based attenuation experiments did not include ENO1 re-expression rescue or gain-of-function validation; therefore, future studies should further confirm the specificity of ENO1-mediated effects using complementary rescue and overexpression approaches.

In summary, this study, through integrative computational modeling, multi-omics profiling, and preliminary *in vitro* validation, suggests that ENO1 is a candidate mediator associated with BaP-related gastric cancer progression. Future investigations are warranted to validate the functional relevance of this relationship *in vivo*, to elucidate the precise molecular events linking BaP exposure to ENO1 upregulation using more refined genetic and biochemical tools, and to explore its biological and clinical significance in exposure-annotated patient populations.

## 5 Conclusion

This study integrated network toxicology with multi-omics analyses to investigate candidate molecular mediators through which the environmental carcinogen benzo [a]pyrene (BaP) may be associated with gastric cancer progression. Through exploratory prioritization and preliminary validation, ENO1 was identified as a candidate target associated with BaP-related malignant phenotypes. By integrating evidence from expression analyses, single-cell transcriptomics, docking-based structural prediction, and *in vitro* experiments, we propose a working model in which ENO1 may contribute to BaP-associated gastric cancer progression through effects on tumor cell behavior and microenvironment-associated cellular states. Overall, these findings provide an initial framework for understanding how BaP exposure may be linked to gastric cancer progression beyond previously reported pathways, while supporting ENO1 as a candidate mediator for further investigation. Further mechanistic, *in vivo*, and clinical studies are needed to determine the precise role of ENO1 in this context.

## Data Availability

The datasets presented in this study can be found in online repositories. The names of the repository/repositories and accession number(s) can be found in the article/Supplementary Material.

## Supplementary material

SUPPLEMENTARY FIGURE S1 External validation of the five prioritized candidate genes in TCGA-STAD. **(A)** ROC analysis of ENO1, GPT, COL4A1, ADH7, and CA9 in TCGA-STAD. **(B)** Expression differences of the five candidate genes between normal and tumor tissues. ROC analysis was used to assess expression-based separability rather than clinical diagnostic performance. **P < 0.01, ***P < 0.001.

SUPPLEMENTARY FIGURE S2 HGC-27 validation of BaP-associated ENO1 regulation and functional phenotypes. **(A,B)** Transwell assay showing BaP-enhanced migration of HGC-27 cells. **(C,D)** Colony formation assay showing BaP-enhanced clonogenic capacity. **(E)** qRT-PCR analysis of ENO1 expression in siNC, siNC + BaP, siENO1, and siENO1 + BaP groups. **(F,G)** Colony formation assay showing reduced clonogenic capacity after ENO1 knockdown. *P < 0.05, ***P < 0.001, ****P < 0.0001.
