# Supplementary material for: An integrated network toxicology and multi-omics study identifies ENO1 as a candidate mediator in benzo[a]pyrene-related gastric cancer progression
Source: Front Pharmacol. 2026 Jun 29;17:1857843. doi: 10.3389/fphar.2026.1857843 (PMC13357143; doi:10.3389/fphar.2026.1857843)
Supplement: Supplementary file 1 [file Supplementaryfile1.docx]

**Supplement materials**

**Table S1. Sequence information used in this study**

| Gene Name | Sequence | | OD |
| --- | --- | --- | --- |
|  | sense（5'-3'） | antisense（5'-3'） |  |
| ENO1-Homo-1215 | GUCAUGGUGUCUCAUCGUUTT | AACGAUGAGACACCAUGACTT | 2.00 |
| ENO1-Homo-986 | GUCUAUCGAAGAUCCCUUUTT | AAAGGGAUCUUCGAUAGACTT | 2.00 |
| ENO1-Homo-430 | AGUUUGGUGCGAACGCCAUTT | AUGGCGUUCGCACCAAACUTT | 2.00 |
| Negative control | UUCUCCGAACGUGUCACGUTT | ACGUGACACGUUCGGAGAATT | 2.00 |

**Table S2. Information on antibodies used in this study**

| Antibody | Cat. No. | WB | Specificity |
| --- | --- | --- | --- |
| N-cadherin | 22018-1-AP | 1：2000 | Rabbit |
| E-cadherin | 20874-1-AP | 1：2000 | Rabbit |
| Vimentin | 10366-1-AP | 1：2000 | Rabbit |
| β-actin | 20536-1-AP | 1：2000 | Rabbit |
| GAPDH | 10494-1-AP | 1：5000 | Rabbit |
| ENO1 | 11204-1-AP | 1：2000 | Rabbit |

**Table S3. qPCR primers used in this study**

| Name |  | Primer sequence |
| --- | --- | --- |
| ENO1 | Forward | 5’-AAAGCTGGTGCCGTTGAGAA-3’ |
|  | Reverse | 5’-GGTTGTGGTAAACCTCTGCTC -3’ |
| β-actin | Forward | 5’-GAGAAAATCTGGCACCACACC -3’ |
|  | Reverse | 5’-GATAGCACAGCCTGGATAGCA -3’ |
