# Supplementary figures and images for: An integrated network toxicology and multi-omics study identifies ENO1 as a candidate mediator in benzo[a]pyrene-related gastric cancer progression
Source: Front Pharmacol. 2026 Jun 29;17:1857843. doi: 10.3389/fphar.2026.1857843 (PMC13357143; doi:10.3389/fphar.2026.1857843)

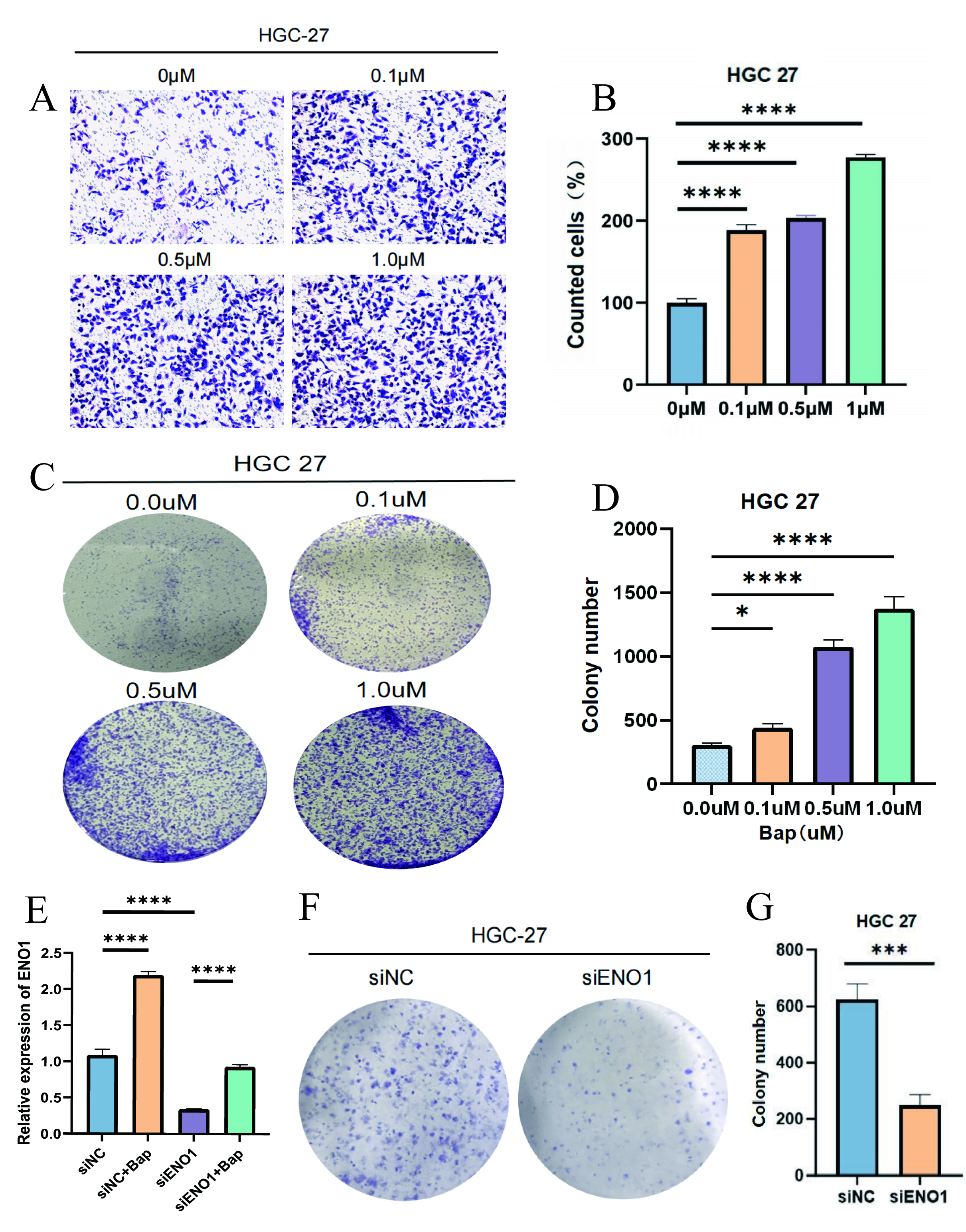

Supplement: Supplementary file 2 [file Image2.TIF]

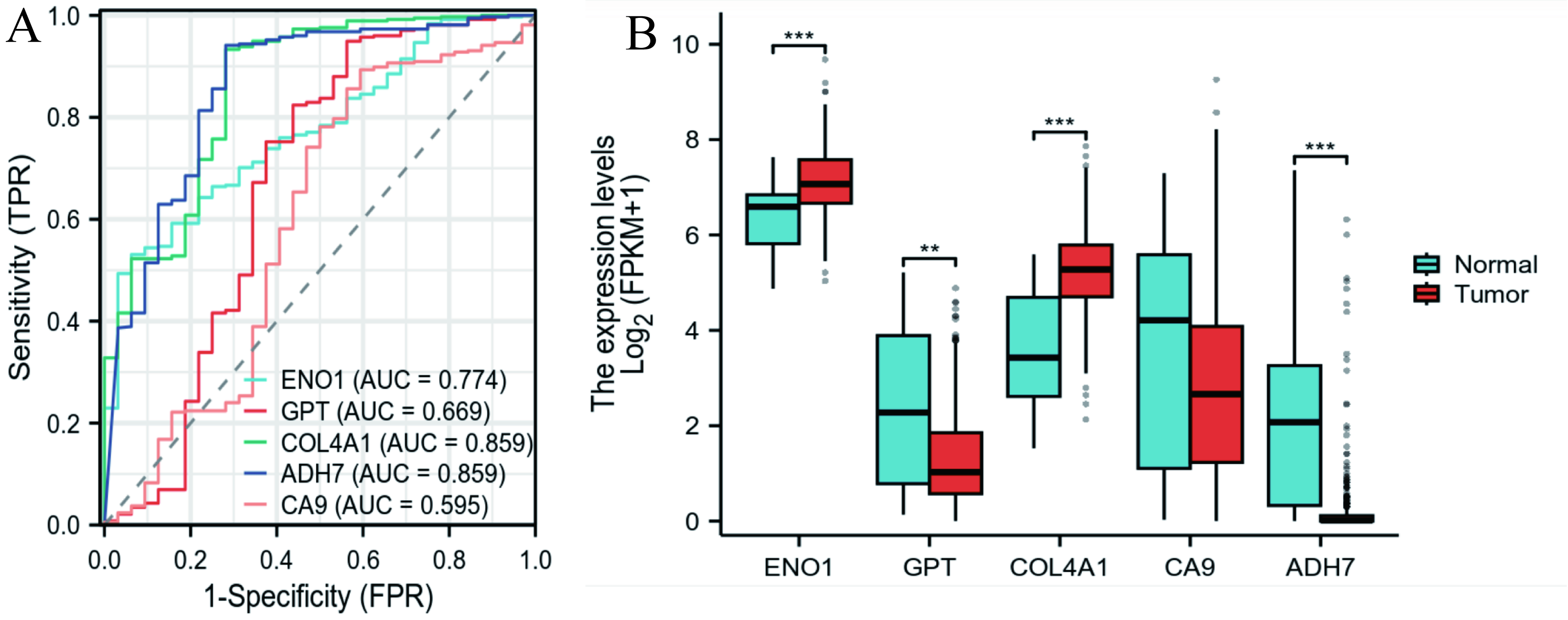

Supplement: Supplementary file 3 [file Image1.TIF]
